# Supplementary material for: A Likelihood Approach for Real-Time Calibration of Stochastic Compartmental Epidemic Models
Source: PLoS Comput Biol. 2017 Jan 17;13(1):e1005257. doi: 10.1371/journal.pcbi.1005257 (PMC5240920; doi:10.1371/journal.pcbi.1005257)
Supplement: S1 File — (TAR.GZ) [file pcbi.1005257.s014.tar.gz › HSPH_Online-SI-Revision/IPoi09/n100-mild/n100-mild_BENCH2_table-8.pdf]

1	newly 3 weeks spec	1329.	3320.08	{2259., 4886.}	166.485	0.731412	newly 3 weeks cumul	2670.	10 573.4	{7700., 14 489.}	312.625	0.782597	newly 5 weeks spec	2409.	2157.83	{698., 4686.}	57.3059	0.604722	newly 5 weeks cumul	6876.	15 549.3	{12 401., 20 846.}	142.785	0.599229	newly 60 weeks cumul	33 899.	19 608.3	{13 007., 28 726.}	42.1566	0.498872
2	newly 3 weeks spec	1754.	4858.24	{1250., 6251.}	189.731	0.47491	newly 3 weeks cumul	4109.	12 848.3	{2221., 19 461.}	228.085	0.527451	newly 5 weeks spec	2989.	4426.65	{2160., 6342.}	63.3775	0.213518	newly 5 weeks cumul	9407.	22 249.9	{8376., 26 607.}	146.135	0.398566	newly 59 weeks cumul	41 142.	35 867.4	{27 496., 41 228.}	12.96	0.0643328
3	newly 3 weeks spec	1224.	7822.64	{5393., 10 015.}	547.49	0.830014	newly 3 weeks cumul	2588.	20 557.	{10 477., 27 171.}	702.838	0.912938	newly 5 weeks spec	2853.	5474.09	{1985., 9550.}	107.899	0.318525	newly 5 weeks cumul	7485.	33 167.4	{28 077., 38 520.}	351.369	0.68081	newly 62 weeks cumul	35 853.	42 796.8	{35 560., 48 294.}	21.7743	0.0845179
4	newly 3 weeks spec	1047.	12 837.	{3867., 22 176.}	1126.08	1.01189	newly 3 weeks cumul	1848.	17 795.7	{4861., 33 473.}	862.972	0.893896	newly 5 weeks spec	3916.	20 711.7	{15 458., 25 263.}	428.898	0.715679	newly 5 weeks cumul	7888.	60 253.	{36 408., 73 289.}	663.857	0.870424	newly 66 weeks cumul	49 382.	86 452.	{83 002., 91 525.}	75.1081	0.240677
5	newly 3 weeks spec	1604.	8521.73	{2928., 10 946.}	431.28	0.68708	newly 3 weeks cumul	3273.	18 664.5	{4998., 28 052.}	470.556	0.697921	newly 5 weeks spec	2839.	8261.23	{6027., 10 131.}	190.991	0.45536	newly 5 weeks cumul	8315.	36 145.4	{19 455., 42 647.}	334.701	0.622623	newly 62 weeks cumul	37 686.	53 254.8	{48 910., 56 189.}	41.312	0.149774
6	newly 3 weeks spec	1697.	4903.87	{3682., 6060.}	188.973	0.448974	newly 3 weeks cumul	3762.	13 528.2	{7397., 17 078.}	259.601	0.535905	newly 5 weeks spec	3382.	3765.	{1769., 5123.}	30.682	0.130715	newly 5 weeks cumul	9658.	21 855.7	{18 483., 25 309.}	126.296	0.348784	newly 60 weeks cumul	39 815.	29 928.6	{22 153., 34 661.}	24.8308	0.128153
7	newly 3 weeks spec	621.	4752.85	{734., 9795.}	668.178	0.756279	newly 3 weeks cumul	1186.	8657.65	{1226., 20 685.}	634.541	0.713621	newly 5 weeks spec	2262.	8081.58	{4763., 11 684.}	257.276	0.529174	newly 5 weeks cumul	4678.	23 584.7	{8111., 36 447.}	404.162	0.650826	newly 65 weeks cumul	38 409.	44 623.2	{38 732., 51 437.}	16.4168	0.062837
8	newly 3 weeks spec	1914.	3971.22	{421., 6239.}	131.093	0.395787	newly 3 weeks cumul	4004.	9298.	{887., 17 590.}	157.784	0.428427	newly 5 weeks spec	3108.	4793.61	{1490., 6567.}	68.7849	0.249093	newly 5 weeks cumul	9610.	18 620.4	{3165., 26 680.}	112.466	0.351902	newly 59 weeks cumul	33 244.	40 523.8	{33 532., 43 773.}	22.3435	0.0863004
9	newly 3 weeks spec	1331.	6472.33	{818., 11 965.}	400.631	0.645014	newly 3 weeks cumul	2488.	10 984.4	{1234., 20 888.}	359.602	0.614568	newly 5 weeks spec	3831.	11 844.1	{5492., 14 511.}	213.95	0.482682	newly 5 weeks cumul	8579.	32 904.3	{9067., 48 215.}	291.236	0.561956	newly 64 weeks cumul	48 106.	67 345.9	{61 712., 75 837.}	39.9947	0.144262
10	newly 3 weeks spec	2104.	1642.64	{356., 3883.}	63.8999	0.400706	newly 3 weeks cumul	4740.	7070.82	{2579., 13 086.}	74.2558	0.237534	newly 5 weeks spec	3114.	729.273	{155., 2194.}	76.5808	0.883819	newly 5 weeks cumul	10 319.	8946.55	{2887., 17 676.}	51.8805	0.281355	newly 58 weeks cumul	38 689.	9856.27	{2994., 21 026.}	74.5244	0.696929
11	newly 3 weeks spec	890.	3199.	{6., 5794.}	296.162	0.883178	newly 3 weeks cumul	1597.	7264.68	{28., 15 192.}	390.785	0.838369	newly 5 weeks spec	2552.	3848.58	{0., 6164.}	86.3203	0.782934	newly 5 weeks cumul	5799.	14 801.8	{49., 24 319.}	191.096	0.736848	newly 62 weeks cumul	44 256.	29 171.2	{49., 39 346.}	34.0853	0.453221
12	newly 3 weeks spec	1981.	4528.78	{788., 6561.}	144.259	0.40461	newly 3 weeks cumul	4456.	11 630.5	{1504., 17 839.}	178.081	0.447951	newly 5 weeks spec	3330.	4643.56	{2043., 6171.}	55.5989	0.203275	newly 5 weeks cumul	10 451.	21 067.7	{5474., 28 167.}	115.066	0.347068	newly 58 weeks cumul	44 615.	38 327.5	{28 430., 43 416.}	14.0928	0.0703328
13	newly 3 weeks spec	1116.	3922.08	{1455., 7255.}	260.093	0.523023	newly 3 weeks cumul	2037.	7467.2	{2383., 13 758.}	275.737	0.533254	newly 5 weeks spec	3104.	6758.08	{4110., 9576.}	124.353	0.350062	newly 5 weeks cumul	7098.	19 848.	{10 071., 32 241.}	187.314	0.445988	newly 63 weeks cumul	40 666.	45 555.3	{29 695., 56 673.}	20.8134	0.0852326
14	newly 3 weeks spec	1519.	4345.87	{393., 9640.}	225.021	0.726289	newly 3 weeks cumul	2463.	7971.13	{681., 19 559.}	264.225	0.703906	newly 5 weeks spec	4569.	6918.97	{3594., 10 218.}	68.2432	0.500641	newly 5 weeks cumul	9906.	20 954.7	{5626., 34 395.}	135.069	0.588886	newly 65 weeks cumul	48 448.	41 429.5	{38 274., 51 228.}	16.5234	0.359341
15	newly 3 weeks spec	1182.	2476.35	{197., 5632.}	145.555	0.476896	newly 3 weeks cumul	2013.	4455.89	{337., 10 647.}	157.757	0.489977	newly 5 weeks spec	3065.	6289.51	{989., 11 118.}	127.923	0.450591	newly 5 weeks cumul	7143.	14 950.6	{1714., 30 430.}	136.591	0.467739	newly 62 weeks cumul	48 233.	77 677.3	{59 510., 89 816.}	64.6823	0.309579
16	newly 3 weeks spec	1433.	3773.47	{1169., 5252.}	172.803	0.419501	newly 3 weeks cumul	3454.	9697.21	{1990., 14 838.}	195.232	0.45394	newly 5 weeks spec	2363.	3789.89	{2365., 4712.}	64.8395	0.213479	newly 5 weeks cumul	7772.	17 466.9	{6127., 23 432.}	133.233	0.357997	newly 58 weeks cumul	32 590.	33 462.7	{30 045., 38 583.}	9.33948	0.0401051
17	newly 3 weeks spec	933.	5689.26	{1524., 8603.}	524.153	0.898893	newly 3 weeks cumul	1663.	11 895.2	{2264., 23 357.}	630.278	0.946222	newly 5 weeks spec	2774.	6068.61	{2165., 8620.}	133.635	0.533689	newly 5 weeks cumul	6118.	24 403.9	{12 027., 32 710.}	312.429	0.790287	newly 64 weeks cumul	46 517.	36 362.3	{34 665., 39 437.}	21.83	0.330596
18	newly 3 weeks spec	223.	20 665.	{0., 46 132.}	9252.82	2.67174	newly 3 weeks cumul	346.	29 880.1	{0., 71 594.}	8621.82	2.69461	newly 5 weeks spec	1979.	3765.18	{0., 10 029.}	179.989	2.08715	newly 5 weeks cumul	3142.	45 978.	{0., 90 305.}	1449.33	2.70294	newly 68 weeks cumul	44 313.	47 051.6	{0., 91 324.}	92.1797	2.54879
19	newly 3 weeks spec	1287.	3971.22	{421., 6239.}	224.735	0.502426	newly 3 weeks cumul	2744.	9298.	{887., 17 590.}	256.791	0.523936	newly 5 weeks spec	2837.	4793.61	{1490., 6567.}	82.785	0.27991	newly 5 weeks cumul	7629.	18 620.4	{3165., 26 680.}	159.36	0.420362	newly 59 weeks cumul	38 174.	40 523.8	{33 532., 43 773.}	9.69886	0.0408066
20	newly 3 weeks spec	216.	1722.99	{0., 3990.}	831.144	2.09427	newly 3 weeks cumul	340.	2003.82	{0., 4405.}	622.7	2.02914	newly 5 weeks spec	1268.	5430.92	{0., 25 921.}	456.989	2.47144	newly 5 weeks cumul	2188.	11 804.2	{0., 57 519.}	569.679	2.45715	newly 68 weeks cumul	40 762.	43 942.9	{0., 92 455.}	101.952	2.64571
21	newly 3 weeks spec	570.	12 360.7	{0., 39 089.}	2148.54	2.30419	newly 3 weeks cumul	880.	15 275.5	{0., 45 297.}	1716.18	2.31168	newly 5 weeks spec	2390.	11 241.7	{0., 29 621.}	450.365	2.24272	newly 5 weeks cumul	4463.	42 852.7	{0., 88 687.}	940.176	2.56684	newly 68 weeks cumul	44 593.	50 681.4	{0., 92 883.}	93.9447	2.42397
22	newly 3 weeks spec	1853.	5144.08	{2468., 7238.}	189.576	0.472983	newly 3 weeks cumul	4544.	13 162.2	{4920., 19 236.}	202.64	0.487512	newly 5 weeks spec	2912.	5094.32	{1923., 7026.}	87.1071	0.274518	newly 5 weeks cumul	9679.	23 626.2	{13 566., 30 765.}	154.805	0.416197	newly 58 weeks cumul	43 141.	41 903.1	{31 472., 49 465.}	11.8369	0.0564658
23	newly 3 weeks spec	936.	4625.17	{1189., 8360.}	399.27	0.610804	newly 3 weeks cumul	1680.	9233.92	{2049., 18 935.}	456.448	0.638718	newly 5 weeks spec	2751.	6972.71	{4380., 8876.}	157.905	0.403303	newly 5 weeks cumul	5966.	22 342.2	{8710., 34 983.}	279.136	0.536112	newly 63 weeks cumul	45 926.	48 338.2	{44 256., 54 939.}	6.49151	0.0265944
24	newly 3 weeks spec	678.	3268.93	{5., 8372.}	420.092	0.961704	newly 3 weeks cumul	1172.	4112.27	{23., 10 346.}	294.079	0.82344	newly 5 weeks spec	2554.	16 636.1	{34., 26 591.}	575.877	1.17225	newly 5 weeks cumul	4980.	30 565.	{47., 58 640.}	542.108	1.0858	newly 67 weeks cumul	42 361.	78 135.5	{44 304., 93 547.}	104.168	0.746221
25	newly 3 weeks spec	1220.	3773.47	{1169., 5252.}	215.824	0.471093	newly 3 weeks cumul	2798.	9697.21	{1990., 14 838.}	257.046	0.516528	newly 5 weeks spec	2397.	3789.89	{2365., 4712.}	62.7912	0.208542	newly 5 weeks cumul	7013.	17 466.9	{6127., 23 432.}	156.014	0.392507	newly 58 weeks cumul	41 718.	33 462.7	{30 045., 38 583.}	19.7884	0.0986138
26	newly 3 weeks spec	1422.	3320.08	{2259., 4886.}	150.147	0.706933	newly 3 weeks cumul	2792.	10 573.4	{7700., 14 489.}	295.323	0.766427	newly 5 weeks spec	3228.	2157.83	{698., 4686.}	53.671	0.658582	newly 5 weeks cumul	8236.	15 549.3	{12 401., 20 846.}	105.447	0.533912	newly 60 weeks cumul	43 633.	19 608.3	{13 007., 28 726.}	55.0608	0.608501
27	newly 3 weeks spec	1429.	2815.43	{754., 5872.}	121.201	0.349838	newly 3 weeks cumul	3015.	5705.16	{1412., 12 336.}	118.108	0.353668	newly 5 weeks spec	2878.	5509.98	{2160., 8700.}	105.403	0.31771	newly 5 weeks cumul	7889.	15 338.5	{4684., 28 813.}	113.282	0.331513	newly 58 weeks cumul	41 619.	80 116.3	{69 478., 89 315.}	92.4994	0.282569
28	newly 3 weeks spec	1395.	2818.	{1371., 4742.}	118.961	0.624837	newly 3 weeks cumul	3462.	9740.92	{7042., 13 553.}	198.033	0.816414	newly 5 weeks spec	2612.	1505.75	{282., 3883.}	63.6198	0.774531	newly 5 weeks cumul	8023.	13 404.5	{8250., 19 990.}	83.7426	0.629485	newly 59 weeks cumul	37 337.	15 957.8	{8500., 26 974.}	57.26	0.798404
29	newly 3 weeks spec	1153.	4148.38	{1288., 6972.}	266.77	0.502285	newly 3 weeks cumul	2468.	8834.67	{2263., 16 430.}	268.662	0.492028	newly 5 weeks spec	2181.	5572.48	{4188., 7027.}	159.628	0.406444	newly 5 weeks cumul	6228.	19 505.	{9102., 29 168.}	218.236	0.467079	newly 61 weeks cumul	30 741.	41 364.5	{38 184., 43 726.}	34.5582	0.12842
30	newly 3 weeks spec	1065.	2499.94	{14., 5514.}	173.797	0.607728	newly 3 weeks cumul	1901.	5205.81	{69., 12 726.}	212.53	0.594839	newly 5 weeks spec	3590.	3623.56	{11., 5320.}	41.4781	0.572188	newly 5 weeks cumul	7402.	12 015.4	{77., 21 841.}	101.908	0.523743	newly 63 weeks cumul	47 995.	30 686.2	{26 713., 36 828.}	36.0638	0.333038
31	newly 3 weeks spec	1303.	3130.5	{295., 6249.}	167.003	0.438231	newly 3 weeks cumul	2596.	6790.72	{544., 15 250.}	189.653	0.458882	newly 5 weeks spec	2456.	4528.78	{788., 6561.}	102.624	0.33805	newly 5 weeks cumul	6882.	15 290.7	{1753., 26 840.}	145.007	0.4085	newly 60 weeks cumul	37 472.	41 987.7	{37 431., 44 074.}	12.2125	0.0495371
32	newly 3 weeks spec	2005.	2354.	{852., 4686.}	60.7436	0.248173	newly 3 weeks cumul	4624.	9050.09	{4680., 13 374.}	96.2783	0.262802	newly 5 weeks spec	3092.	1146.45	{227., 3092.}	63.7157	0.621554	newly 5 weeks cumul	10 135.	11 839.2	{5189., 19 351.}	47.8845	0.190975	newly 58 weeks cumul	42 820.	13 478.2	{5452., 24 396.}	68.5236	0.564403
33	newly 3 weeks spec	116.	18 364.9	{0., 43 881.}	15 808.8	2.57355	newly 3 weeks cumul	183.	24 206.2	{0., 63 427.}	13 204.4	2.58855	newly 5 weeks spec	1068.	7056.64	{0., 19 363.}	636.734	2.08568	newly 5 weeks cumul	1629.	47 173.6	{0., 88 513.}	2871.86	2.61633	newly 68 weeks cumul	33 313.	50 967.4	{0., 91 883.}	128.996	2.33866
34	newly 3 weeks spec	1093.	3462.3	{656., 7410.}	237.825	0.589673	newly 3 weeks cumul	1962.	6011.39	{994., 14 036.}	231.122	0.539761	newly 5 weeks spec	3687.	8910.61	{4371., 14 155.}	155.694	0.522908	newly 5 weeks cumul	7846.	20 979.4	{7041., 36 611.}	182.986	0.528016	newly 64 weeks cumul	44 642.	66 136.6	{48 059., 92 351.}	52.731	0.259798
35	newly 3 weeks spec	20.	25 480.8	{0., 47 686.}	127 368.	2.95056	newly 3 weeks cumul	142.	51 328.	{0., 84 429.}	36 110.5	2.85636	newly 5 weeks spec	625.	1191.82	{0., 2407.}	162.051	1.48087	newly 5 weeks cumul	970.	59 185.	{0., 93 062.}	6065.55	2.60286	newly 68 weeks cumul	40 997.	59 412.8	{0., 93 323.}	108.92	2.01866
36	newly 3 weeks spec	277.	6314.3	{0., 22 515.}	2277.44	2.23526	newly 3 weeks cumul	444.	7880.31	{0., 26 208.}	1774.66	2.23894	newly 5 weeks spec	1470.	10 243.3	{0., 28 559.}	691.82	2.45711	newly 5 weeks cumul	2688.	28 727.3	{0., 83 232.}	1064.09	2.62164	newly 68 weeks cumul	33 226.	46 871.8	{0., 92 895.}	131.069	2.67143
37	newly 3 weeks spec	1497.	3602.83	{2578., 5053.}	157.337	0.719652	newly 3 weeks cumul	2865.	10 404.3	{5069., 14 629.}	279.689	0.691401	newly 5 weeks spec	3432.	2818.	{1371., 4742.}	39.6173	0.539243	newly 5 weeks cumul	8555.	16 542.3	{12 894., 21 772.}	109.988	0.50649	newly 61 weeks cumul	44 446.	22 759.3	{18 153., 29 632.}	48.7935	0.512157
38	newly 3 weeks spec	250.	22 129.5	{0., 47 307.}	8839.8	2.7097	newly 3 weeks cumul	547.	34 174.2	{0., 76 892.}	6235.56	2.75922	newly 5 weeks spec	1505.	2477.2	{0., 5946.}	157.737	2.05691	newly 5 weeks cumul	2547.	46 593.7	{0., 90 394.}	1817.36	2.77354	newly 68 weeks cumul	38 637.	47 300.6	{0., 92 296.}	110.423	2.63628
39	newly 3 weeks spec	1338.	11 403.5	{4561., 16 269.}	756.388	0.898904	newly 3 weeks cumul	2487.	19 872.2	{6626., 31 083.}	703.519	0.855339	newly 5 weeks spec	3912.	11 291.6	{6864., 16 630.}	188.64	0.433368	newly 5 weeks cumul	8892.	45 092.9	{33 958., 52 926.}	408.423	0.692781	newly 65 weeks cumul	39 846.	58 227.1	{53 708., 62 935.}	46.1304	0.164067
40	newly 3 weeks spec	586.	8891.54	{0., 16 864.}	1453.04	1.65472	newly 3 weeks cumul	886.	13 230.4	{0., 26 687.}	1428.99	1.66709	newly 5 weeks spec	3300.	7458.57	{0., 15 891.}	162.879	1.1364	newly 5 weeks cumul	5752.	31 620.	{0., 45 939.}	485.437	1.52278	newly 67 weeks cumul	45 872.	36 587.6	{0., 48 549.}	23.4127	1.03588
41	newly 3 weeks spec	397.	10 159.3	{0., 31 310.}	2538.95	2.23409	newly 3 weeks cumul	582.	12 110.8	{0., 36 213.}	2060.62	2.17706	newly 5 weeks spec	2106.	12 099.8	{0., 30 323.}	554.373	2.2588	newly 5 weeks cumul	3564.	40 748.2	{0., 85 132.}	1123.07	2.44634	newly 68 weeks cumul	48 915.	51 273.5	{0., 92 477.}	83.519	2.27624
42	newly 3 weeks spec	795.	3268.1	{613., 6460.}	327.167	0.565395	newly 3 weeks cumul	1426.	5147.04	{878., 10 104.}	281.747	0.525593	newly 5 weeks spec	2995.	10 488.1	{4853., 14 688.}	258.418	0.53684	newly 5 weeks cumul	5937.	22 159.9	{7528., 36 709.}	282.975	0.554529	newly 65 weeks cumul	44 109.	70 385.9	{56 120., 88 171.}	60.6742	0.198924
43	newly 3 weeks spec	339.	18 829.8	{0., 44 784.}	5552.51	2.75221	newly 3 weeks cumul	542.	25 897.2	{0., 69 499.}	4776.06	2.79337	newly 5 weeks spec	1978.	3639.51	{0., 10 474.}	181.999	2.34281	newly 5 weeks cumul	3344.	41 606.6	{0., 89 455.}	1242.21	2.90465	newly 68 weeks cumul	47 323.	42 667.7	{0., 91 150.}	88.1625	2.8877
44	newly 3 weeks spec	386.	2870.57	{0., 7910.}	677.258	1.11647	newly 3 weeks cumul	600.	3677.98	{0., 10 958.}	547.717	1.04912	newly 5 weeks spec	2455.	14 824.3	{0., 26 033.}	534.492	1.31797	newly 5 weeks cumul	4090.	27 056.8	{0., 59 564.}	592.297	1.29033	newly 67 weeks cumul	47 913.	68 800.1	{0., 91 950.}	72.1613	0.931643
45	newly 3 weeks spec	1342.	7264.91	{3191., 9823.}	450.156	0.75828	newly 3 weeks cumul	2662.	16 051.9	{5727., 25 076.}	511.837	0.778848	newly 5 weeks spec	2863.	7136.32	{3593., 9978.}	157.767	0.424883	newly 5 weeks cumul	7410.	31 010.9	{19 963., 38 417.}	327.102	0.66088	newly 63 weeks cumul	42 303.	46 114.4	{41 776., 48 823.}	10.194	0.041927
46	newly 3 weeks spec	594.	4096.66	{470., 7757.}	603.458	0.875644	newly 3 weeks cumul	1181.	7216.34	{689., 14 971.}	527.101	0.794933	newly 5 weeks spec	2667.	8430.34	{1986., 10 597.}	230.934	0.643204	newly 5 weeks cumul	5207.	22 216.1	{3601., 34 823.}	341.823	0.710946	newly 64 weeks cumul	48 956.	52 592.5	{51 733., 54 419.}	13.221	0.161407
47	newly 3 weeks spec	763.	1888.53	{385., 4607.}	170.537	0.387573	newly 3 weeks cumul	1478.	2998.58	{557., 7465.}	132.703	0.349801	newly 5 weeks spec	3193.	7313.22	{1946., 12 880.}	141.379	0.361812	newly 5 weeks cumul	6436.	14 369.8	{3688., 28 989.}	140.217	0.355898	newly 64 weeks cumul	48 321.	83 549.5	{70 585., 93 040.}	72.9052	0.234934
48	newly 3 weeks spec	1223.	2557.93	{507., 4415.}	129.558	0.365406	newly 3 weeks cumul	2426.	5979.29	{1060., 11 373.}	166.347	0.406423	newly 5 weeks spec	3101.	3152.79	{1123., 4393.}	29.3246	0.156735	newly 5 weeks cumul	7512.	12 080.9	{2838., 19 226.}	83.8706	0.289127	newly 59 weeks cumul	44 170.	31 690.9	{27 559., 34 944.}	28.2524	0.145521
49	newly 3 weeks spec	820.	8901.4	{2331., 14 057.}	990.551	0.958502	newly 3 weeks cumul	1602.	15 921.6	{3294., 27 633.}	899.928	0.906558	newly 5 weeks spec	2523.	10 029.3	{7275., 12 752.}	297.514	0.583168	newly 5 weeks cumul	5593.	36 893.4	{21 172., 47 283.}	559.636	0.786368	newly 65 weeks cumul	35 659.	53 224.2	{52 307., 54 206.}	49.2588	0.173883
50	newly 3 weeks spec	648.	10 438.7	{2421., 15 589.}	1510.92	1.13879	newly 3 weeks cumul	1146.	17 295.1	{3127., 31 925.}	1409.17	1.0837	newly 5 weeks spec	2869.	10 773.9	{5992., 14 430.}	275.528	0.547781	newly 5 weeks cumul	5616.	41 252.9	{25 274., 48 774.}	634.561	0.855157	newly 66 weeks cumul	40 068.	51 679.5	{49 710., 51 890.}	28.9794	0.109546
